# Resolution of inflammation: targeting GPCRs that interact with lipids and peptides^☆^

**DOI:** 10.1016/j.drudis.2014.06.023

**Published:** 2014-08

**Authors:** Jenna L. Cash, Lucy V. Norling, Mauro Perretti

**Affiliations:** The William Harvey Research Institute, Barts and The London School of Medicine and Dentistry, Charterhouse Square, London EC1M 6BQ, UK

## Abstract

There is a growing appreciation of the important role of resolution mediators in the successful termination of the inflammatory response. Here, we discuss the potential importance of the lipid and peptide proresolving mediators, in particular the resolvins and chemerin-derived peptides, which mediate their effects through specific G protein-coupled receptors (GPCRs).

## Introduction

The typical result of inflammation is removal of harmful stimuli, such as pathogens, followed by resolution; that is, the restoration of affected tissues to their normal structural and functional state. Until recently, it was thought that resolution of the acute inflammatory response was a passive process; it is now evident that endogenous anti-inflammatory and proresolving pathways exist to control the generation of an appropriate inflammatory response and its resolution [1]. The obvious implication of this is that chronic inflammatory pathologies could be in part explained by a ‘failure to resolve’ and, hence, be a consequence, again at least partly, to the absence or malfunction of one or more proresolving pathways. Improved understanding of endogenous anti-inflammatory systems, in part through identification of novel resolution mediators and receptors, could establish novel paradigms that not only explain the pathology (e.g. inadequate activation of proresolving mechanisms and pathways), but also underpin the development of novel drugs that can promote inflammatory resolution, perhaps in concert with the endogenous pathways of the body [2].

**Figure 1:**
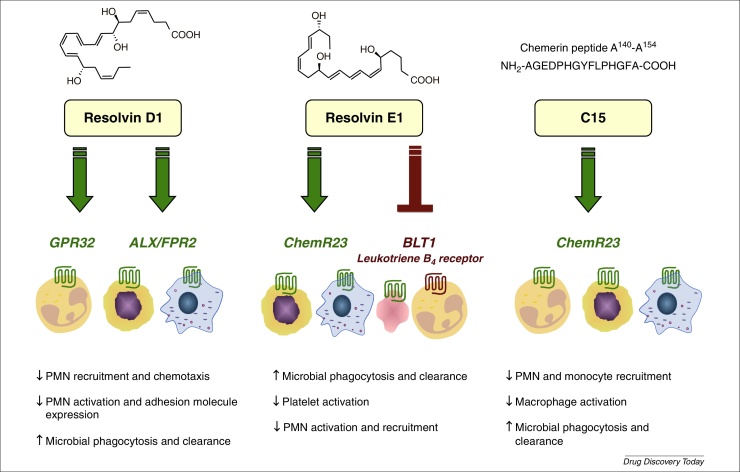
Key cellular actions of resolvins and the chemerin peptide C15. Resolvins act in a stereospecific manner on multiple cell types via specific G protein-coupled receptors (GPCRs) to limit neutrophil (PMN) activation and recruitment and to stimulate nonphlogistic macrophage phagocytosis. Both RvD1 and RvE1 act at two GPCRs, RvD1 signals via ALX/FPR2 and an orphan receptor GPR32 on human leukocytes, whereas RvE1 acts as an agonist at ChemR23 and as a partial agonist on the LTB_4_ receptor (BLT1), thus competing with LTB_4_ for binding (reviewed in [27]). The chemerin peptide C15 is also known to signal via ChemR23 to reduce PMN and monocyte recruitment and limit macrophage activation. Abbreviations: ALX/FPR2, lipoxin A4 receptor/annexin-A1 receptor/formyl peptide-like 2; LTB_4_, leukotriene B_4_; RvD1, resolvin D1; RvE1, resolvin E1;.

A diverse array of factors has a role in inflammatory resolution, including gaseous mediators (H_2_S [3]); a purine (adenosine [4]); acetylcholine release from the vagal nerve [5]; a protease inhibitor [secretory leukocyte protease inhibitor (SLPI) [6]]; lipids {lipoxins [7], resolvins [8], protectins [9], maresins [10], and cyclopentenone prostaglandins [15-deoxy-delta-12,14-prostaglandin J2 (15d-PGJ2)] [2]}; proteins (annexin A1 [11]); and peptides (annexin, melanocortin and chemerin-derived peptides 12, 13, 14, 15, 16) (Table 1, Table 2). In this noncomprehensive review, we focus on a subset of membrane anti-inflammatory GPCRs as effectors of resolution, ChemR23 (CMKLR1), GPR32 and FPR2/ALX, which transduce the proresolving signals of chemerin peptides, resolvin E1 (RvE1) and resolvin D1 (RvD1) (Fig. 1).

**Table 1:** A selection of proresolving mediators and their receptors

**Resolution mediator (abbreviation)**	**Synonyms**	**Class**	**Receptor(s)**	**Refs**
**Annexin A1 (AnxA1)**	Lipocortin A1		FPR2/ALX	11, 12, 55
**Galectin 1 (Gal1)**	Galaptin, LGALS1	Protein	CD7, CD43, CD45, integrins, CD2, CD3	56, 57, 58
**Galectin 9 (Gal9)**	LGALS9		TIM-3	56, 59
**Ac2-26**			FPR1, FPR2/ALX	30, 60
**Alpha-melanocortin-stimulating hormone (αMSH)**	α-Melanotropin	Peptide	MC3R	61, 62
**Chemerin15 (C15)**			ChemR23/CMKLR1	16, 49
**Lipoxin A4 (LXA4)**			FPR2/ALX, GPR32	7, 28, 63
**Resolvin D1 (RvD1)**			FPR2/ALX, GPR32	28, 64
**Resolvin D2 (RvD2)**			?	20, 26
**Resolvin E1 (RvE1)**		Lipid	ChemR23, BLT1	52, 65, 66
**Maresins**			?	[10]
**Protectin D1**			?	9, 67
**15-Deoxy-prostaglandin J2 (15d-PGJ2)**	Cyclopentenone prostaglandin		PPARγ	[68]

**Table 2:** Ligands for selected resolution receptors

**Receptor**	**Ligands**	**Refs**
**BLT1**	LTB4	[69]
	RvE1	[52]
**ChemR23**	Chemerin (TIG2, RARRES2)	16, 38
	C15^a^	16, 49
	RvE1	52, 66
**FPR1**	fMLF	[70]
	Mitochondrial formyl peptides	[71]
	Ac2-26	13, 72
**FPR2**	AnxA1	[73]
	CCL23	[74]
	Humanin	75, 76
	SHAAGtide	[77]
	Ac2-26	13, 72
	SAA	[78]
	uPAR	[79]
	PrP (Prion protein)	[80]
	LL-37 (Cathelicidin)	[81]
	Temporin	[82]
	Lipoxin A4	[83]
	WKYMVm	84, 85

a Receptor specificity shown indirectly through use of receptor-knockout cells and mice.

## Proresolving lipid agonists: resolvins

We first focus on the role that resolvins, as examples of proresolving lipids, have in inflammatory resolution. Omega-3 polyunsaturated fatty acids (PUFA) are known to be beneficial for health. Indeed, population studies suggest that these lipids have a preventative effect in rheumatoid arthritis (RA), with lower prevalence observed in the Japanese and Inuit population, who consume large amounts of oily fish rich in omega-3 PUFA. In corroboration, clinical studies have revealed that dietary supplementation with omega-3 PUFA is efficacious in reducing joint pain, morning stiffness, and nonsteroidal anti-inflammatory drugs (NSAID) usage in patients with RA [17]. Additionally, consumption of omega-3 PUFA has favorable effects for cardiovascular health [18], which can become compromised in patients with RA. However, the mechanisms by which omega-3 PUFAs exert their beneficial effects has not yet been fully explored.

Recently, a new genus of autacoids was identified in resolving exudates that exert potent, protective properties and control the duration and magnitude of an inflammatory response. These include the lipoxins from arachidonic acid and the omega-3-derived resolvins, protectins, and maresins [19]. Here, we focus on two of the resolvins (resolution-phase interaction products) with identified target receptors, namely RvE1 and RvD1, which are enzymatically biosynthesized from omega-3 eicosapentaenoic acid and docosahexaenoic acid, respectively.

Resolvins exert potent anti-inflammatory and proresolving actions not only in acute inflammatory models, but also in models of chronic disease, including diabetes, sepsis, retinopathy, asthma, atherosclerosis, and periodontitis. RvD1, D2, and E1 also exhibit anti-infective actions, enhancing the containment, killing, and clearance of bacteria to promote catabasis 20, 21, 22. Furthermore, resolvins help maintain vascular homeostasis; RvE1 counter-regulates platelet activation [23] and decreases platelet-derived growth factor-stimulated vascular smooth muscle cell activation [24]. Additionally, RvD2 stimulates vasoprotective prostacyclin and nitric oxide release from vascular endothelial cells [20]. Resolvins were recently identified as potent analgesics; 17R-RvD1 (100 ng intraperitoneally twice daily) is antihyperalgesic, reducing hind paw withdrawal frequency in a model of adjuvant-induced arthritis, which was associated with decreased tumor necrosis factor (TNF)-α and interleukin (IL)-1β levels within the paw [25]. Most recently, RvD1, D2, and E1 were documented as endogenous inhibitors for transient receptor potential vanilloid 1 (TRPV1) and TRP ankyryn 1 (TRPA1) currents; these receptors contribute to inflammatory pain via peripheral and central sensitization, thus explaining the analgesic actions of resolvins [26].

The bioactions of resolvins are mediated via specific GPCRs (Fig. 1). RvE1 acts as an agonist at two GPCRs, namely ChemR23 and as a partial agonist on the leukotriene B_4_ (LTB_4_) receptor (BLT1), thus competing with LTB_4_ for binding (reviewed in [27]). RvD1 is also known to act via two GPCRs, which were identified and validated using a GPCR/beta-arrestin coupled system, the lipoxin A4 (LXA_4_) and annexin-A1 receptor [formyl peptide-like 2 (FPR2)/ALX] and an orphan receptor GPR32 on human leukocytes [28] (Fig. 1). Specific binding experiments revealed that RvD1 binds with high affinity (*K*_d_ = 0.2 nM) to human neutrophils. RvD1 binding could be partially displaced (approximately 60%) by LXA_4_, whereas no competition was observed with the annexin peptide Ac2-12, conferring independent peptide and/or lipid binding sites. Receptors for other resolvins are yet to be determined, but are likely to be high-affinity GPCRs based on their potency, stereoselective actions and because their actions can be blocked with the selective Gα_i_-coupled GPCR inhibitor, pertussis toxin 20, 26.

Transgenic mice overexpressing human FPR2/ALX exhibited reduced neutrophil infiltration in zymosan peritonitis [29] and mice lacking the murine homologue receptor displayed an exacerbated response to arthritogenic serum [30], further supporting a protective role for this receptor in inflammation. Indeed, increased levels of the proresolving mediator LXA_4_ and FPR2/ALX are detected in human pathologies, including RA [31] and acute post-streptococcal glomerulonephritis [32], suggesting that protective mediators and their receptors are may be operative within inflammatory settings to aid resolution. Therefore, endogenous lipid mediators are temporally and spatially biosynthesized to regulate actively resolution by acting on their specific GPCRs, which initiates anti-inflammatory and proresolving signals to terminate inflammation. However, when these endogenous counter-regulatory circuits fail, inflammation perpetuates, as observed in pathologies such as atherosclerosis [33] and periodontitis [34], which are associated with chronic low-grade inflammation.

## Proresolving peptide agonists: chemerin and its peptides

Chemerin is a chemoattractant protein less commonly known as retinoic acid receptor responder (RARRES2) and tazarotene-induced gene-2 (TIG-2). Chemerin is found in the circulation and in inflammatory exudates including ascitic and synovial fluid 35, 36. Secreted as an inactive precursor, pro-chemerin undergoes C-terminal proteolytic cleavage by serine proteases to generate the active chemoattractant protein. These enzymes include those of the coagulation (factor VII) and fibrinolytic (plasmin) cascades, and those derived post-neutrophil degranulation (elastase and cathepsin G) 35, 36, 37. Chemerin acts as a plasmacytoid dendritic cell, natural killer cell, and macrophage chemoattractant 38, 39, 40. The chemotactic effects of chemerin are mediated through the GPCR ChemR23, although chemerin can also bind to GPR1 and chemokine (C–C motif) receptor-like 2 (CCRL2) [chemokine receptor on activated macrophages (CRAM)] 41, 42. The binding sites of chemerin on each of its receptors have yet to be described and it is currently unknown where, or indeed if, chemerin peptides bind to the aforementioned chemerin receptors, although the chemerin-derived peptide C15 clearly mediates its effects through ChemR23. With the exception of the ability of chemerin to induce a calcium flux response in GPR1-transfected cells, its functional relevance as a GPR1 ligand *in vitro* or *in vivo* is unknown [43]. The situation with respect to CCRL2 is a little clearer. CCRL2, similar to the Duffy antigen for chemokine receptor (DARC) and D6, is not thought to be a signaling receptor. Indeed, CCRL2 binds but does not internalize chemerin, thus increasing local chemerin concentrations available to interact with ChemR23 [44]. CCRL2^−/−^ mice display reduced tissue swelling, suggesting a role for the receptor in edema; however, CCRL2 has several identified ligands, including chemokine (C–C motif) ligand 5 and 19 (CCL5 and CCL19); thus, it is unclear whether the phenotype described is the result of changes in chemerin sequestration [45].

**Figure 2:**
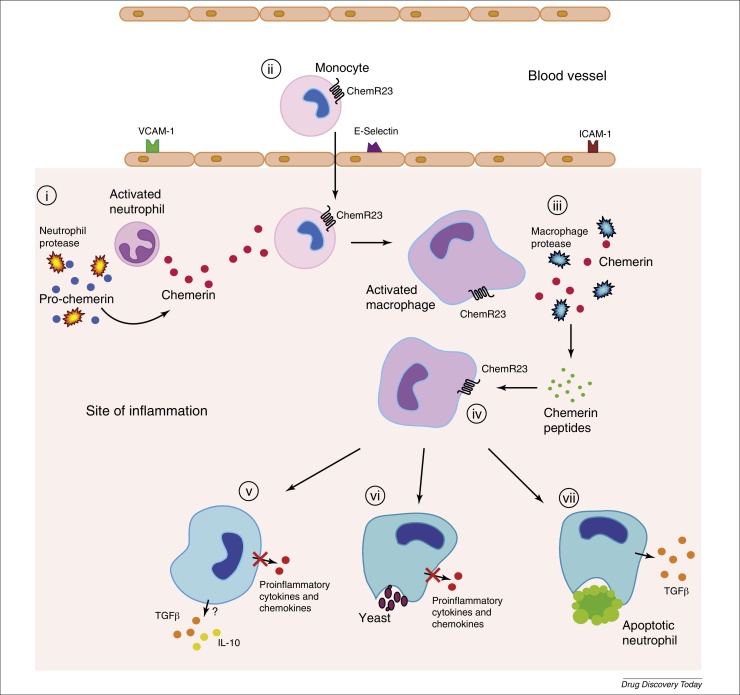
Pathways and effects for chemerin, chemerin peptides and ChemR23 in inflammation. (i) Pro-chemerin is cleaved by proteolytic enzymes released upon neutrophil degranulation at the inflammatory site, generating the potent chemoattractant chemerin 35, 36, 38. (ii) Chemerin engages ChemR23 on circulating monocytes and tissue macrophages (MФ), recruiting these cells to the inflamed site [38]. (iii) Activated MФs release proteolytic enzymes to eliminate and digest invading organisms; however, they also serve to cleave chemerin to generate (iv) potent anti-inflammatory peptides, capable of engaging ChemR23 to reprogram activated monocyte-derived MФs toward an anti-inflammatory and/or proresolving phenotype. (v) The expression of proinflammatory mediators by MФs is repressed and anti-inflammatory and wound repair cytokines, including interleukin (IL)-10 and tumor growth factor (TGF)-β are induced. Chemerin peptides (e.g. C15; see Fig. 1) promote efficient clearance of pathogens (vi) and apoptotic cells (vii) at the inflammatory site, thereby aiding restoration of normal tissue structure and function.

Chemerin was initially described as a transcript upregulated by the anti-inflammatory psoriasis drug, tazarotene, in skin raft cultures [46] and induced by the anti-inflammatory compounds 1,25 dihydroxyvitamin D3 and dexamethasone [47] in an osteoblast cell line, suggesting that it has beneficial roles in inflammation. Indeed, chemerin can undergo further proteolysis of the C terminus by cysteine proteases, primarily macrophage-derived cathepsins, to generate peptides endowed with either anti-inflammatory or antimicrobial properties 16, 48. The 15-amino acid chemerin-derived peptide C15 (AGEDPHGYFLPGQFA) (Figure 1, Figure 2) inhibits macrophage activation in picomolar concentrations and, in the context of the acute inflammatory response, C15 suppresses neutrophil and monocyte recruitment (up to 65%) and inhibits proinflammatory cytokine (TNFα, IL-1β, IL-12 p40, and IL-6) and chemokine [CCL2 (JE) and CXCL1 (KC)] expression [16]. Importantly, C15 promotes the nonphlogistic clearance of apoptotic neutrophils and microbial particles from the inflammatory milieu, thus contributing to the resolution of inflammation [49] (see Fig. 2 for a dynamic scheme of the chemerin–C15–ChemR23 axis). Chemerin can also be cleaved by cathepsin L and K to generate antimicrobial peptides capable of reducing growth of a spectrum of bacteria, including *Escherichia coli*, *Klebsiella pneumonia* [48]. Furthermore, chemerin administration in a lipopolysaccharide (LPS)-induced lung inflammation model resulted in dampened neutrophil recruitment and inflammatory cytokine expression indicative of *in vivo* proteolysis to afford generation of the anti-inflammatory and proresolving species [50]. Collectively, these data describe a unique protein requiring proteolytic processing to activate its latent chemoattractant properties and further proteolysis to release separate antimicrobial and anti-inflammatory and/or proresolving peptides.

The anti-inflammatory and proresolving effects of C15 are mediated by ChemR23 because ChemR23^−/−^ cells and mice are unresponsive to the peptide, whereas neutralization of endogenous chemerin species results in exacerbation of peritonitis 16, 49. Furthermore, LPS-induced lung inflammation is also exacerbated in ChemR23^−/−^ mice [50], whereas in a model of viral pneumonia, ChemR23^−/−^ animals exhibited higher mortality, delayed viral clearance, and increased neutrophil recruitment [51]. Collectively, these studies demonstrate an important anti-inflammatory and proresolving role for chemerin peptides and ChemR23 in acute inflammation.

Binding of chemerin and RvE1 to ChemR23 has been demonstrated using radiolabelled agonists; however, conclusive binding studies have yet to be performed for C15 38, 52. One group has, surprisingly, not reproduced any of the data obtained with RvE1 or C15. In particular, Luangsay *et al.* failed to show displacement of chemerin from its binding site on ChemR23 by RvE1 or C15 and concluded that they are not ligands for ChemR23 [50]. It is established for other GPCRs, such as formyl peptide like 2 (FPR2), that the proinflammatory serum amyloid A (SAA) binds to a distinct site on the receptor to the anti-inflammatory protein AnxA1 13, 53; thus, one cannot conclude that lack of chemerin displacement by RvE1 and/or C15 means that these mediators are not ligands for the receptor. Indeed, the complexity is emerging and it is now accepted that these receptors rarely function as one ligand–one signal receptors. Given that the binding sites for C15, RvE1, and chemerin within ChemR23 have yet to be mapped, we propose three potential scenarios to explain the apparent discrepancies: (i) the anti-inflammatory molecules C15 and RvE1 bind to a distinct, and as yet, unidentified site on ChemR23 to the chemoattractant chemerin to exert their opposing effects on inflammation; (ii) RvE1 and/or C15 displace chemerin from ChemR23 but interact with different GPCR residues, triggering different signaling pathways; or (iii) ligand-biased heterodimerization of ChemR23 with another, possibly related, GPCR could allow binding of chemerin peptides and RvE1 to a receptor that is dimerized with ChemR23 but still produces ChemR23 downstream effects. This scenario has been demonstrated for FPR2/ALX, which can heterodimerize with Leukotriene B4 receptor (BLT1) [54] and can also convey both pro-inflammatory signals and have lipid, protein, and peptide ligands. With continued research, we predict that more examples of peptido- and lipid-based agonists sharing the same receptor will be unveiled and perhaps could become a paradigm for GPCRs.

## Concluding remarks

The discovery that specific GPCRs can transduce signals from both lipids and peptides is not only a novel aspect in receptor biology that is likely to become more common in the years ahead, but is also endowed with important opportunities for drug discovery. We postulate that nature has economized to make use of the same receptor to convey proresolving, inhibitory, and buffering signals by short-lived lipids and also by peptides and/or proteins, with longer half-lives (hours versus minutes), and often generated at later stages of inflammation. One example that emerges from this approach to research is that of ChemR23, a specific GPCR that signals effects of RvE1 and C15. We conclude that a better understanding of the pharmacology of these receptors, especially in chronic inflammatory settings, could guide innovative drug discovery programs aimed at capitalizing the fundamental actions of these effectors of resolution. This has already begun to happen, with a stable isopropyl ester analog of RvE1, RX-10045 (Resolvyx Pharmaceuticals) proving efficacious in a Phase II clinical trial to treat the signs and symptoms of dry eye (Clinicaltrials.gov identifier: NCT00799552), and with C15 being an ideal candidate for canonical structure–activity relation studies to develop novel anti-inflammatory therapeutics.
